# Vascular bioactivation of nitroglycerin: reaction mechanism revealed by crystal structure of aldehyde dehydrogenase-2

**DOI:** 10.1186/2050-6511-13-S1-A37

**Published:** 2012-09-17

**Authors:** Barbara S Lang, Antonius CF Gorren, Gustav Oberdorfer, M Verena Wenzl, Cristina M Furdui, Leslie B Poole, Bernd Mayer, Karl Gruber

**Affiliations:** 1 Department of Pharmacology and Toxicology, Institute of Pharmaceutical Sciences, University of Graz, 8010 Graz, Austria; 2 Institute of Molecular Biosciences, University of Graz, 8010 Graz, Austria; 3 Department of Internal Medicine, Section of Molecular Medicine, Wake Forest School of Medicine, Winston-Salem, NC 27157, USA; 4 Department of Biochemistry, Wake Forest School of Medicine, Winston-Salem, NC 27157, USA

## Background

Aldehdyde dehydrogenase-2 (ALDH2) is essential for the detoxification of ethanol in the liver but also catalyzes vascular bioactivation of nitroglycerin (glycerol trinitrate, GTN) to its active metabolite nitric oxide (NO), which causes vasodilation through accumulation of cyclic GMP. The clinical use of GTN as a vasodilator is hampered by loss of efficacy after prolonged treatment, and there is strong evidence that this results from mechanism-based inactivation of ALDH2 by GTN. The precise mechanism of the ALDH2/GTN reaction as well as the identity of the inactivated enzyme species is still elusive.

## Methods

To address these issues, we have determined the crystal structure of an ALDH2 mutant in complex with GTN. In addition, the 3D structures of a reaction intermediate and of the GTN-inactivated enzyme were resolved.

## Results

GTN is bound to the active site by hydrogen bonds to the so-called oxyanion hole, to Gln268 to Ser301 and by hydrophobic interactions. It is held in a position ideal for the nucleophilic attack of the active site Cys302. After this nucleophilic attack, a thionitrate is formed and 1,2-glyceryl dinitrate is released. This thionitrate was observed in the second crystal structure. It is in a position similar to the corresponding atoms of GTN and stabilized by hydrogen bonds to the oxyanion hole. Finally, the structure of the inactivated enzyme species determined to a resolution of 1.7 Å agrees well with mass spectrometric results, suggesting that exposure to GTN causes in oxidation of the active site Cys302 to sulfinic acid.

## Conclusions

Our results provide important new insights into the complex mechanism of ALDH2-catalyzed GTN bioactivation and the development of nitrate tolerance.

